# The Role of Sexual Selection in the Evolution of Chemical Signals in Insects

**DOI:** 10.3390/insects5020423

**Published:** 2014-06-18

**Authors:** Sandra Steiger, Johannes Stökl

**Affiliations:** 1 Institute of Experimental Ecology, University of Ulm, Albert-Einstein-Allee 11, 89081 Ulm, Germany; 2 Institute of Zoology, University of Regensburg, Universitätstraße 31, 93053 Regensburg, Germany

**Keywords:** sexual selection, mate choice, pheromone, communication, chemical signal

## Abstract

Chemical communication is the most ancient and widespread form of communication. Yet we are only beginning to grasp the complexity of chemical signals and the role they play in sexual selection. Focusing on insects, we review here the recent progress in the field of olfactory-based sexual selection. We will show that there is mounting empirical evidence that sexual selection affects the evolution of chemical traits, but form and strength of selection differ between species. Studies indicate that some chemical signals are expressed in relation to an individual’s condition and depend, for example, on age, immunocompetence, fertility, body size or degree of inbreeding. Males or females might benefit by choosing based on those traits, gaining resources or “good genes”. Other chemical traits appear to reliably reflect an individual’s underlying genotype and are suitable to choose a mating partner that matches best the own genotype.

## 1. Introduction

Sexual selection is one of the strongest evolutionary forces, and consequently a central topic in evolutionary biology that has continuously received high attention over the last decades. Recent progress in the field of sexual selection appears to be mainly based on studies on visual and acoustic ornaments (e.g., [1,2]). Although research examining the role of sexual selection in the evolution of chemical signals is still underrepresented, many studies dealing with sexual selection on olfactory traits have been accumulated in recent years and there is now growing appreciation that sexual selection can shape chemical signals [3]. Nevertheless, it remains uncertain how chemical signals are elaborated. Does sexual selection affect the signal’s compositional complexity or the quantities of expression? Are chemical signals condition-dependent and which mechanisms sustain signal honesty? Do animals choose chemical traits that indicate good genes or genetic compatibility? Chemical communication is the predominant mode of communication in insects [4], and therefore most data come from the insect world [5]. The substances involved differ highly in their molecular structures and physico-chemical properties. Some can only be perceived upon contacts, some are employed at a short range, and yet others act over long distances. Although it appears that in the majority of species studied today several components are subjected to sexual selection, the exact numbers are often unknown. Here, we review the recent advances in the field of sexually selected chemical traits focusing on insects. Our aim is not to provide an exhaustive list of studies, we rather focus on some important studies that—so we believe—progressed our understanding since the comprehensive review of Johansson and Jones [3] on “the role of chemical communication in mate choice”. We also emphasize that we do not review mate choice in general, *i.e.*, we do not address chemical signals that are solely involved in species or sexual recognition, but refer to recent reviews on this topic [6,7]. 

In this review we will summarize the evidence for sexual selection on chemical traits from evolutionary quantitative genetics and selection experiments and we will address the honesty and condition dependence of chemical signals. Additionally, we will discuss whether chemical signals reflect good genes and/or genes that are a good fit and shortly review the role of sensory exploitation in the evolution of sexually selected chemical traits.

## 2. Evidence from Evolutionary Quantitative Genetics and Selection Experiments

To understand sexual selection as an evolutionary process different theoretical and empirical approaches have been adopted in the past [8]. With one of them, the evolutionary quantitative genetic approach, the microevolutionary potential of sexually selected traits can be described and therefore short-term evolution can be predicted [9,10,11]. There is an increasing number of studies that analyze form and strength of sexual selection on chemical traits based on the formal statistical framework provided by Lande and Arnold [12] and some of them measure both sexual selection gradients as well as genetic (co-) variances via the additive genetic variance-covariance matrix (G-matrix). Only in the latter case, we can determine the rate and direction in which a population may respond to a given selection regime as we have knowledge about the genetic variation for the traits under sexual selection. However, although quantitative genetics appears to be a growing field within the framework of sexual selection and chemical communication, the studies are currently confined—at least to our knowledge—to the genus *Drosophila* and to crickets. Consequently, there is a strong bias of the chemical traits measured, as all studies to date focused on cuticular hydrocarbons (CHCs). 

### 2.1. Estimating Strength and Form of Sexual Selection

In general, as long as some measure of fitness of individuals is available, selection gradients can be estimated for any quantifiable trait in any taxon [12,13]. With regard to sexual selection and chemical traits, the common method is to randomly draw individuals from a population, let them choose mating partners (here, the protocols differ slightly) and afterwards analyze the chemical traits (CHCs) of the rejected (unattractive) and accepted (attractive) mates (e.g., [14]). Individuals that obtain a mating are assigned an absolute fitness score of 1, the unsuccessful individuals of 0. Selection gradients evaluate the strength and form of selection and are usually calculated following Lande and Arnold [12] with some further adaptation. All studies published to date found evidence for sexual selection acting on CHCs, however, form and strength differ depending on the studied organism and sex. In *Drosophila serrata*, for example, female preference primarily resulted in directional (linear) sexual selection on male CHCs, whereas male preference resulted in stabilizing (non-linear) and correlational selection on female CHCs [15]. Overall, 8 CHCs were analyzed, and the targets of linear selection were four components, 5,9-C25diene, 2-Me-C28, 5,9-C29diene and 2-Me-C30 (in a different study, all 8 components were under strong directional selection; [16]), while the targets of stabilizing selection were two substances, 9-C26ene and 2-Me-C26. There are two explanations for the stabilizing selection on females. Individual males could show directional preference for female CHC, but different males in opposing directions, leading to stabilizing selection on the population level. Alternatively, males may truly prefer females with an intermediate CHC profile. A preference for an intermediate phenotype could for example be explained by a resource trade-off for females between CHCs and vital functions [15]. Although it remains unknown, why the selection regimes and the substances under significant selection differ between the sexes, the result might explain the evolution of sexual dimorphism in this species [15]. 

Other studies in *Drosophila* found significant directional sexual selection on male CHCs: in *D. bunnanda* 6 out of 15 CHCs were under linear sexual selection [17]; in *D. subquinaria* and *D. recens*, the picture is not as clear cut, as not the individual CHCs were assessed but principal components [18]. In *D. simulans*, on the other hand, CHCs were subject to complex patterns of directional, stabilizing, disruptive and correlational sexual selection, but the non-linear gradients were estimated to be stronger than the linear ones [19]. Also in the two cricket species studied to date, a more complex pattern of sexual selection on CHCs emerged. In the Australian field cricket *Teleogryllus oceanicus*, there was significant stabilizing and disruptive selection on male as well as female CHCs [20,21,22], in the sagebrush cricket *Cyphoderris strepitans*, analyses revealed both significant linear and nonlinear selection on male CHCs [23]. In both cricket species, stabilizing and disruptive selection resulted in a saddle-shaped fitness surface, a surface that seems to be characteristic for other sexually selected traits in crickets as well, including male song traits [24,25], morphological characters [26] and the chemical composition of nuptial food gifts [27]. It is also noteworthy that the study on sagebrush cricket measured sexual selection on CHCs of free-living individuals [23]. However, we are far from understanding, why certain CHCs or combination of CHCs are more attractive to females or males than others. If we examine more closely which chemical substances or combinations of substances are targeted by sexual selection, we find no clear picture, at least not yet. In the sagebrush cricket, for example, there appears to be complex interplay between the total amount of CHCs and specific CHC combinations. Disruptive selection acted most strongly on the total amount coupled with selection on specific combinations of mono-, di- and trimethylalkanes [23]. The other issue we have to keep in mind, is that these studies are merely correlative. Although all studies measured significant sexual selection on CHCs, we cannot rule out that other traits are targeted by sexual selection and CHCs simply correlated with those traits. However, in the Australian field cricket, Simmons *et al.* [22] found significant selection on both CHC profile and courtship song parameters, but there was no correlation between the attractiveness of a male’s song and his CHC profile, indicating that CHCs are directly affected by sexual selection.

**Box 1: Glossary**

**Sexual selection.** Selection arising from variation in the ability to obtain a mate. Sexual selection leads to evolution of features that enhance mating success. Sexual selection takes two major forms: intersexual selection (‘mate choice’) and intrasexual selection (‘male-male competition’).

**Selection gradient (β).** The gradient of a regression of fitness on trait value.

**Selection gradient, quadratic (γ).** The coefficient of a regression of squared deviation of a trait against fitness. Negative values indicate stabilizing selection, which reduces the variance of the trait.

**Directional selection.** Favors one extreme phenotype over the mean or other extreme.

**Stabilizing selection.** Favors intermediate trait values by selection against extreme phenotypes. Stabilizing selection is supposed to decrease the genetic variance.

**Disruptive selection.** Selects against an average phenotype. A population under disruptive selection would show phenotypes of both extremes.

**Correlational selection.** Selection for optimal character combinations. When two or more traits affect fitness in an interactive way, selection is correlational.

**Cuticular hydrocarbons (CHCs).** CHCs often form a thin waxy layer on an insect’s epicuticle consisting of a mix of saturated and unsaturated, straight chain and branched hydrocarbons with a chain length from approx. 10 to 40 carbon atoms. Usually the mix contains more than 20 compounds. In many insect species CHCs are involved in recognition and communication processes, e.g., in nestmate recognition in social insects or as a close range sex or courtship pheromone.

**Lek paradox.** Directional selection by persistent female choice for particular male traits should erode genetic variance in male traits so that females will no longer profit from discriminating among males based on these traits. Yet, females continually display strong preferences for males with relatively elaborate traits.

### 2.2. Genetic Variance and Microevolutionary Potential

As outlined above, measuring sexual selection gradients will not necessarily give an accurate representation of how chemical traits will evolve. The response to sexual selection will depend on genetic variation in trait expression, more precisely on how much genetic variation there is in the direction of selection. The less the genetic variation aligns with the direction of sexual selection, the higher the genetic constraints for trait evolution. However, there are only a small number of studies that measured both sexual selection gradients and genetic (co-)variance with regard to chemical traits and our knowledge is currently limited to the fruit fly *Drosophila*. Investigations of *D. serrata* [28,29] and *D. bunnanda* [17] show that there is generally ample additive genetic variance in male CHCs assessed during female mate choice, however, genetic variance is low for the specific combination of CHCs under directional selection. In fact, the genetic variance was roughly orthogonal, approximately 75° in *D. serrata* [28,29] and 88° in *D. bunnanda* [17], to the direction of sexual selection, indicating that sexual selection has depleted genetic variance in CHC combinations under selection and that further evolution will be slow (e.g., [30]). This leads automatically to a central question in the framework of sexual selection, also known as the “lek paradox”: Why do females, in the absence of nuptial gifts, continue to choose if there are so few genetic benefits of choice? First of all, not all studies found a lack of additive genetic variance in the direction of selection on CHCs. In *D. simulans* the genetic constraints was much lower, with an angle ranging between 19° and 30° [19]. Secondly, the studies on *D. serrata* and *D. bunanda* show that there is substantial genetic variance for the combination of traits that are not aligned with the direction of sexual selection [17,28,29]. Therefore changes in the direction of sexual selection, for example due to novel environments, can maintain genetic variance. Indeed, if subjected to novel environments, *D. serrata* females changed their preferences for certain CHCs, *i.e.*, CHCs that were unattractive in the ancestral environment increased in attractiveness in the novel environment [31]. This in turn might feed back on the evolution of these chemical traits. 

### 2.3. Experimental Evolution Studies

Some few studies on *Drosophila* have conducted selection experiments with regard to chemical signaling and there are several things to learn from them. Hunt *et al.* [32], for example, subjected *D. pseudoobscura* to elevated (six males were housed with one female) or relaxed sexual selection (one single male was housed with one female) for 82 generations and measured the evolutionary response on CHCs. The lines from the intense sexual selection treatment were characterized by an overall higher quantity of CHCs than the relaxed sexual selection lines, indicating that sexual selection leads to the evolution of increased CHC production (or relaxed sexual selection to a decreased CHC production). In addition, the intensity of sexual selection also affected the relative composition of CHCs, revealing that both, absolute quantities as well as CHC composition are targeted by sexual selection [32]. However, as the authors emphasized themselves, using this design of experimental evolution, it cannot be determined, which mechanism of sexual selection, male-male competition or female mate choice or both are responsible for the result. A different selection study was conducted on *D. serrata* by Hine *et al.* [33]. The authors first determined which combination of male CHCs were preferred by female flies (*i.e.*, estimated selection gradients) and then implemented artificial selection for the preferred CHC combination and for a CHC combination that was under weak sexual selection. The preferred CHC traits responded to selection and three out of eight CHCs evolved in the direction of sexual selection leading to a higher mating success compared to control lines or the lines selected for the combination that was less preferred by the females. However, already after seven generations an evolutionary limit was reached. A depletion of genetic variance could be excluded as possible cause of the evolutionary limit, as the authors could even observe an increase in genetic variance in response to artificial selection. When Hine *et al.* [33] relaxed the selection on the preferred chemical traits, about 50% of the previous selection response was lost already after 5 generations, indicating that antagonistic natural selection had played a major role in stopping the evolution of male attractiveness. This result also confirms other studies that have shown that the evolution of CHCs is characterized by an antagonism of sexual selection and natural selection and not all CHC combination favored by sexual selection are favored by natural selection (e.g., [34]).

## 3. Honesty and Condition Dependence of Chemical Signals

### 3.1. Honesty and the Costs of Chemical Signaling

According to most models, the aim of mate choice is to select a mate that is in some way superior to the other potential mates, via a process termed “mate assessment”, assuming that this choice translates into more or fitter offspring. Signals used for mate assessment must therefore provide some information on the quality of the sender, beyond species and sex. Signals advertising the quality of the sender need to fulfil three basic requirements to be useful in mate choice. First, they need to vary between individuals, making an assessment and choice possible. Second, the signals should inform about the quality of the sender genuinely, which means the signals should be honest. And third, a sexually selected signal is expected to show a higher additive genetic variance than naturally selected traits [35]. In models trying to explain honest signaling, the handicap principle plays a central role. Proposed by Zahavi [36] and mathematically verified by Grafen [37], the handicap principle states that honest signals need to contain a specifically wasteful cost, the so called *strategic cost*, on top of the basic costs needed to transfer the signal (*efficacy costs*) [38]. The idea is that it costs a high quality male less to make the same signal as a low quality male. However, several alternative models have shown that efficacy costs only and even cost-free (with negligible efficacy costs) honest signaling can evolve (reviewed by [35]). The payment of realized strategic costs (“handicaps”) by the signaler is thus neither necessary for honest signaling, nor a sufficient condition for honest signaling. Theory rather predicts that honesty is not maintained by the realized costs paid by honest signalers at the equilibrium but by the potential costs of cheating [35]. This does not mean that signaling cannot be costly and in most cases honest signaling appears to be costly, but the paradigm “signals must be costly to be honest” is no longer valid. Another problem is that it is usually impossible to separate strategic costs from efficacy costs. Measuring the realized costs, of pheromone production for example, is therefore not a useful approach to test honest signaling.

One experimental approach circumventing these limitations has recently been pointed out by Higham [39]. Forcing individuals experimentally to cheat, thus to exhibit a signal higher than they should according their relative quality, could enable us to identify the potential costs of cheating [39]. Harari *et al.* [40] followed this approach (although not specifically, as the study was conducted two years earlier) in their study on fitness costs of pheromone production in females of the moth *Lobesia botrana*. They could demonstrate that large females have higher amounts of the major pheromone component (*E*,*Z*)-7,9-dodecadienyl acetate in their glands during calling than small females and that males prefer the pheromone signals of large females over those of small females. Exposure of virgin females to the pheromone of conspecific females increased calling time on the first day but significantly reduced lifetime and the number of laid eggs (after mating) in small females, but not in large ones [40]. This result suggests that small females were forced to cheat by perceiving the sex pheromone of other females, as this indicated the presence of other females. Small females invested more energy into pheromone communication than they should, at the cost of reduced lifetime and fecundity. This suggests that in *L. botrana* calling is actually associated with costs and honestly reflects female condition. However, it remains unclear whether the cost lies in the biosynthesis of the pheromone or in the expansion of energy during calling.

### 3.2. Condition Dependence of Chemical Signals

In the previous paragraph we showed that it can be very difficult to demonstrate the honesty of chemical signals in an empirical study. However, there is plenty of evidence that chemical signals can be condition dependent. Hill [41] defined a condition dependent signal trait as “a conspicuous feature of an organism that varies in expression depending on the capacity to withstand environmental challenges”. Three components contribute to condition: the somatic state (stored resources, parasite load, toxins, *etc.*), the genotype (“good genes”, heterozygosity, inbreeding), and the epigenetic state [41]. However, in living organisms these three components are tightly linked and not always distinguishable in an experimental setting. 

In the classic scenario for mate choice females are supposed to choose those males for mating that provide the best direct or indirect (“good genes”) benefit for their offspring. By choosing a male with “good genes” the female can provide its offspring with the best genes, for example for a high resistance to parasites, a low degree of inbreeding, or high fertility. All those benefits can be revealed to the female by chemical signals.

Several recent studies have demonstrated a correlation between the condition of the male or female sender, the chemical signals and mate choice. For example, in the tomato fruit borer moth *Neoleucinodes elegantalis* males prefer the chemical signals of heavier females over those of light females [42]. Similar results have been reported for the mantid *Stagmomantis limbata*. Females fed with a high quality diet attracted more males by olfactory signals than females fed with a low quality diet [43]. The same has been found in *Pseudomantis albofimbriata*, also a praying mantid. Behavioral experiments demonstrated a preference of males based solely on olfactory signals for egg-bearing females over egg-free females. The olfactory signals of the females are thus a reliable indicator of female fecundity [44]. However, in all those studies the chemical signal itself has not been analyzed and experimental evidence whether egg-bearing females produce higher quantities or a qualitative “better” pheromone is missing. In the polyandrous moth *Heliothis virescens* pheromone production of virgin and mated females depends on feeding status, *i.e.*, the hemolymph trehalose concentration [45]. This dependency can be exploited by males of *H. virescens* to assess the sugar resources of a female, thus her quality, using the female produced pheromone as an indicator [46].

Studies on the mealworm beetle *Tenebrio molitor* have demonstrated a connection between immunocompetence and attractiveness of the male sex pheromone. Experimental immune-challenge or infection by a tapeworm significantly reduced the attractiveness of the male sex pheromone to females, but did not affect survival of the males [47,48,49]. Surprisingly, repeated immune-challenge of males increased their attractiveness to females, but also mortality. This indicates a terminal investment of the males on sexual signaling, *i.e.*, pheromone production, at the expense of lacking recovery of the immune system [50,51]. However, these studies did not investigate the chemical composition of the pheromone of immune-challenged males. 

“Good genes” and the degree of inbreeding in an individual are tightly connected. The deleterious effects of consanguineous mating on the quality of an individual are well known and recent studies could demonstrate an effect of inbreeding on sexual signaling as well. Pheromones produced by inbred males of mealworm beetles (*T. molitor*) and the butterfly *Bicyclus anynana* were less attractive to females than the sex pheromones produced by outbred males [52,53]. Interestingly, the attractiveness of the sex pheromone of female *T. molitor* was not affected by inbreeding [52]. 

Finally, sex pheromones can also be an indicator of the male’s age. In the butterfly *Bicyclus anynana* females prefer to mate with older (14 days) males over younger (3 days) males and females take this decision based on the male sex pheromone. The pheromone consists of three components, but only one (hexadecanal) shows a 100% increase in relative amount between young and old males. The total amount of the pheromone, however, does not change with male age. Interestingly, hexadecanal titres showed no heritability, while the other two pheromone components showed a moderate heritability. This indicates that there is no additive genetic variance left to increase hexadecanal titres [54], questioning the evolutionary benefit for females when choosing such a male. Females of the European corn borer *Ostrinia nubilalis* also prefer to mate with older males and also in this species the male pheromones released from hairpencils trigger female decision. The male sex pheromone of *O. nubilalis* consists of four compounds (C16 unsaturated acetates), but only the titers of two change significantly with moth age [55]. Both studies raise the question why females should prefer to mate with older males over younger ones. One possible explanation is that older males might have proven their ability to survive by reaching a certain age but further studies are needed to demonstrate a higher quality of older males.

Only few of the studies mentioned above also investigated in detail how the pheromone amount and composition is affected by the condition of the sender and have verified their results with synthetic mimics of the chemical trait (e.g., [54,55]). Often it is assumed, that high quality senders simply produce higher amounts of the pheromone, but interestingly, in those two studies mentioned above changes in the proportions of the pheromone components were decisive for female preference, not changes in the absolute pheromone amount. Here definitely more research is needed to understand the difference in the pheromone signals between high and low quality senders.

### 3.3. The Mechanistic Link between Condition and Trait

Despite of abundant evidence for condition dependent sex pheromones in many insect species, the mechanistic link between condition and chemical trait is almost always unknown. Hill [41] lists four possible mechanisms that could link the production of a signal with condition (see also [56] for chemical traits). 

Regarding chemical traits, the resource tradeoff hypothesis is perhaps the most evident mechanism. Under this hypothesis, limited resources are traded off between vital pathways and pheromone production. Nevertheless, unambiguous examples are very rare. One example for this hypothesis are defensive pyrrolizidine alkaloids (PAs) used by the arctiid moth *Utetheisa ornatrix*. Larvae sequester PAs from the host plant and retain them through metamorphosis into adulthood [57]. Females choose males based on a PA derived courtship pheromone, hydroxydanaidal (HD). During mating males transfer a spermatophore whose contents (sperm and PAs) are correlated with the HD titer and body size. Females allocate the received PAs to the eggs for protection. Male pheromone titer thus reliably signals a direct benefit the female can get during mating. A new study however, showed that the HD signal produced by males does not change with mating history [58]. The HD titer thus signals original PA content and body size of the male and females choosing this male provide their offspring with genes to sequester protective chemicals. HD is therefore not only important to signal PA content but also to signal the male’s genetic quality. Another example for the resource tradeoff hypothesis has recently been demonstrated in the parasitoid wasp *Nasonia vitripennis*. In *N. vitripennis*, male sex pheromone biosynthesis is directly linked with spermatogenesis by a shared resource: linoleic acid (LA) [59]. Males from LA rich hosts have a higher number of sperm and a higher pheromone titer than males from hosts poor in LA. Females of *N. vitripennis* prefer higher doses of the male pheromone, thereby avoiding matings with sperm depleted males. Because females of *N. vitripennis* mate only once, sperm depletion would result in less daughters and thus in a significantly reduced fitness of the female. Females furthermore prefer hosts rich in LA during oviposition, which increases both the fertility and attractiveness of their sons [60,61]. A third example was found in *Heliothis virescens*. If mated females of this moth are limited in resources, they allocate more carbohydrates to egg maturation, resulting in reduced pheromone production [62]. Using ^13^C-labeling the authors were able to follow the course of fed sugars into egg maturation and sex pheromone. 

The second mechanism suggested by Hill [41] is the mediator hypothesis, under which a regulatory agent that promotes signal production depresses a vital function. In vertebrates, testosterone is such a regulatory agent. This hormone promotes elaborated ornaments but suppresses the immune system [63]. In insects, juvenile hormone is suspected to act in a similar way. As mentioned above, females of the mealworm *T. molitor* prefer the sex pheromone of immunocompetent males. Injection of juvenile hormone into males of *T. molitor* increased the attractiveness of their sex pheromone to females but at the same time reduced their immonocompetence [64]. Therefore, juvenile hormone, similar to testosterone, seems to mediate the tradeoff between sexual advertisement and immune function. 

The third and fourth linking mechanism postulated by Hill [41] are the pathway functionality hypothesis (pheromone production is proportional to a product of a vital pathway) and the shared pathway hypothesis (pheromone production shares a pathway with a vital function). However, none of those has yet been demonstrated in sexually selected chemical traits of insects. 

## 4. Good Genes *versus* Genes that Are a Good Fit

There are two main mechanisms how an indirect benefit can lead to enhanced offspring fitness. The first one is that females prefer males with ornaments that indicate good genes, the second one that they prefer males with genes that are most compatible (see e.g., [65]). The latter requires that females are somehow able to assess their own genotype and compare it to the genetic make-up of their potential partner. Are there any chemoecological studies that have found support for one of the two mechanisms? Certainly, there are many studies that indicate that mate choice is based on an absolute criterion and females of a certain population prefer on average the same quantity or quality of specific substances. Furthermore, some of the studies presented in the previous section assume that the analyzed chemical traits reflect the genetic quality of the mate and hence, are examples for the “good genes” hypothesis. However, none of them have demonstrated that the females really benefit by having superior offspring. Nevertheless, there is one study in *Drosophila serrata* that could show that a female preference for CHCs (based on an absolute criterion) was positively genetically correlated with offspring fitness, indicating that females have gained genetic benefits from their choice [66]. What about genetic compatibility? Whereas there are many studies in vertebrates that have investigated the role of genetic dissimilarity and chemical signals in mate choice [4], studies demonstrating this kind of mate choice are rare in insects. To be able to choose genetically compatible males based on odor traits, the traits have to reliably reflect the genetic background of individuals. This is unlikely, if the pheromone involved in mate choice is based on merely two or three components. Therefore it is not surprising that compatibility studies focus on CHCs as chemical traits, as the cuticular profile of most insects comprises a dozen to tens of components [67] and has the potential to reveal the underlying genetics. The profile of the cockroach *Blatella germanica*, for example, consists of about 25 CHCs, and their relative abundance is linked to the genetic relatedness of individuals [68]. The authors could demonstrate that the cockroaches are able to discriminate between siblings and nonsiblings based on the quantitative differences of CHCs and that they prefer non-siblings as mating partners, presumably to avoid any negative consequences of inbreeding. Also a study in the Australian field cricket *Teleogryllus oceanicus* was able to show that individuals preferentially mate with partners who share more dissimilar CHCs [69]. The similarity in CHCs between mating pairs correlated with their genetic distance, indicating that the chemical traits reflect the underlying genotype of an individual. Simmons *et al.* [22] also argue that the preference for genetic dissimilarity might be the reason for the disruptive selection gradient found in this species, as males with CHC profiles that differed from the average CHC profile have the greatest mating success in this species. The authors found also support for the prediction of Mays and Hill [65] that females may use both an absolute and relative criterion for mate choice. The courtships song of *Teleogryllus oceanicus*, which also plays a significant role in mate choice, is likely a condition-dependent signal of good genes, whereas CHC profiles appears to provide cues to genetic compatibility [22]. 

A study in the spotted cucumber beetle *Diabrotica undecimpunctata howardi* specifically investigated whether females choose immunocompetent or immunocompatible males based on CHCs [70]. They did not find that preferred males had a higher immunocompetence than unpreferred ones. However, the CHC profile of accepted mates was more dissimilar to the CHCs of their female partner than the CHC signature of the rejected mates. In addition, offspring from forced matings had a lower immune response than offspring from matings, in which the females were allowed to choose a partner. Altogether, those results indicate that females choose—based on CHCs—immunocompatible mates and that females benefit from their choice by having offspring with higher immunocompetence.

## 5. Sensory Exploitation in Sexual Selection

Sensory exploitation is thought to be a major selective force in the evolution of chemical communication [71,72]. Under this hypothesis chemical signals evolve to match a pre-existing sensory bias in the receiver. Well established examples of sensory exploitation are deceptive flowers of orchids which mimic the sex pheromones of female to trick males into pollination or the trap flowers of Araceae which mimic the oviposition substrate of flies [73,74,75]. In sex pheromones this evolutionary route has hardly been investigated. One example are males of the European bee wolf *Philanthus triangulum* which produce (*Z*)-11-eicosen-1-ol as putative male sex pheromone [76]. The same substance is a major component of the honeybee alarm pheromone [77] and used by female beewolfs as a chemical cue to locate its prey [78,79]. Male beewolfs might therefore exploit a sensory bias of the females for (*Z*)-11-eicosen-1-ol to attract females. Another example could be the parasitoid wasp *Asobara tabida*. Females of this wasp parasitize larvae of *Drosophila* and are able to use *cis*-vaccenyl acetate, the aggregation pheromone of several *Drosophila* species to locate its hosts [80,81,82]. Additionally, *cis*-vaccenyl acetate is also part of the female sex pheromone of *A. tabida* [83]. A sensory bias for *cis*-vaccenyl acetate could have played an important role in the evolution of the sex pheromone in this species. And in *Leptopilina heterotoma*, also a parasitoid wasp, (−)-iridomyrmecin is used as chemical cue to avoid competition between females and as major component of the female sex pheromone. In this case, the sensory receptors needed for the detection of (−)-iridomyrmecin as a cue might have played a role on the evolution of the female sex pheromone [84]. 

We are convinced that sensory exploitation has played a much bigger role in the evolution of insect sex pheromones than currently recognized. Investigating sex pheromones together with the chemical cues from insect ecology, e.g., in the foraging behavior, might help to support this hypothesis. 

## 6. Conclusions and Future Directions

In this review, we have highlighted the role of sexual selection and mate choice in the chemical communication system of insects. CHCs have proven to be very useful phenotypic traits in the field of evolutionary quantitative genetics. However, while a number of studies have measured multivariate sexual selection acting on chemical signals, none has confirmed these findings with experimental manipulation. Consequently, we are missing experimental proof that females and males actually choose mates based upon the compounds under selection. In a first step extracts should be used instead of the whole animal to exclude other sensory modalities than olfaction, but for a final proof synthetic compounds have to be used. We furthermore want to encourage the assessment of the strength and form of sexual selection in other taxa than *Drosophila* and crickets and also the analysis of compounds with other physico-chemical properties than CHCs. We have also argued that there is plenty of evidence for the condition dependence of male and female sex pheromones. However, we have to emphasize that many studies have measured the attractiveness of sex pheromones by means of behavioral tests, but only in very few cases, the qualitative and quantitative differences in the sex pheromones of senders of varying quality have been investigated by chemical analysis. Consequently, assays with synthetic compounds mimicking a high or a low quality signal are missing. Furthermore, few studies have identified the mechanistic link between condition and chemical trait. As the biosynthetic pathway of some chemical traits might be easier to elucidate than the underlying mechanism leading to the production of visual and acoustic signals, we see a huge potential in chemical traits for future studies that would significantly contribute to our understanding of sexual selection in general.
